# Exploring the characteristics of cancer-centred civil society organisations in Chile: A qualitative study

**DOI:** 10.1371/journal.pone.0315589

**Published:** 2025-05-16

**Authors:** Antonia Roberts, Francisca Vezzani, Báltica Cabieses, Alexandra Obach, Carla Campaña, Manuel A. Espinoza

**Affiliations:** 1 Centro de Salud Global Intercultural (CeSGI), Facultad de Medicina Clínica Alemana, Facultad de Psicología, Universidad del Desarrollo, Santiago, Chile; 2 Center for Cancer Prevention and Control (CECAN), FONDAP 152220002, ANID Chile; 3 Department of Health Sciences, University of York, United Kingdom; 4 Departamento de Salud Pública, Escuela de Medicina, Pontificia Universidad Católica de Chile, Chile; UNIANDES, ECUADOR

## Abstract

**Background:**

In Chile, civil society organisations in health have been historically active in population health and specifically in cancer; they have had an important role in addressing patients’ and families’ necessities. Although they occupy a central role, there is no clarity about who they are, how they are organised, the goals that guide their performance and how they materialize social participation in health. Based on that, this study aimed to explore the characteristics of civil society organisations that work in cancer in Chile and to identify the networks they build with other actors to achieve their goals.

**Materials and methods:**

Qualitative case study using semi-structured online interviews with organisation representatives, politicians, decision-makers and academics related to cancer in Chile. Content analysis was performed, admitting emerging categories from the participants’ narratives.

**Results:**

Three main profiles of organisations were identified: (i) Long-established organisations focused on influencing public policy and decision-making in cancer, (ii) Growing organisations focused on informing and supporting families and patients, (iii) Newly established organisations focused on patient well-being, such as sports activities. Relationships between groups and other actors involve perceived benefits like the growth of the organisations and funding for activities. However, perceived barriers and inequities are also identified, mainly lack of financial resources, competition between organisations and insufficient information.

**Discussion:**

The objectives of civil society organisations in cancer are diverse and reflect multiple ways of practising social participation in health. Tensions generate unequal participation and missed opportunities to promote public health in cancer in Chile. The study highlights the importance of recognising cancer social organisations as essential actors in public health. It is crucial to involve them in formulating and implementing comprehensive responses to maximise the opportunities for progress in this field.

## Background

Social participation in health can be defined as the involvement of the community or specific groups within the population in activities related to their health [1,2]. Involvement can occur at different phases, such as the promotion, prevention and provision of health care, service usage, evaluation of health services, and participation in the decision-making process [2]. Social participation can be conceived and practised in different manners according to the agents involved. From the health system, it is usually linked to orientating the use of services, promoting actions and, in some cases, participating in decision-making processes using formal participation bodies and mechanisms. In civil society organisations and social movements in health, experiences of self-management in health are also identified, as communities create ways to meet their needs in this area independent of the healthcare sector [3], which can involve formal and informal linkages with different actors. Throughout Latin America, local experiences of self-management in health have been observed, and they interact differently with the health system, depending on the specific territory [3].

Historically, in Chile, civil society organisations in health have had a vital role in pressuring the State to introduce improvements in health for the population and in addressing several critical aspects of healthcare needs from the 19th century to the present day through self-management [4–6]. Nowadays, health social organisations also set political agendas and fight to reduce inequities in their areas of interest. The enactment of Law 20.850 on Financial Protection for High-Cost Diagnoses and Treatments (Ricarte Soto Law) is a milestone in this area [7,8], as it succeeded in introducing into Chilean legislation participation spaces in health decision-making, driving the process based on social mobilisation.

Along with the experiences of organised citizenship in health, civil society participation in governance has been repositioned at the institutional level as a critical area of social participation [9]. With the enactment of Law 20.500 in 2011 [10], the right of citizens to participate in public affairs was recognised, and it became relevant to consider them in the State’s decision-making process in its various spheres. Specifically in health, with the 2005 reform that introduced the Law on Explicit Health Guarantees, a window was created for participation in primary healthcare and health services with the creation of the Consultative Councils [10]. The authorities conceived this instance as an opportunity to voice the users’ preferences and expectations and as a transparency initiative for the system to achieve the social legitimisation of the health system to overcome the previously identified citizen dissatisfaction [11]. Currently, the health system continues to move towards establishing citizen participation at the consulting level [10] without creating spaces for meaningful participation beyond the Ricarte Soto Law, nor have any structural advances in the interaction with civil society.

A public health problem that has become a key concern for social health organisations is cancer. Organisations have played a fundamental role in making inequities visible and pushing institutions to advance significantly in managing this disease [12]. In 2018, the National Cancer Plan 2018–2028 was launched, which outlined a comprehensive action plan on the subject and included citizen participation as a central pillar of it [13]. Subsequently, in 2020, the National Cancer Law was enacted [12]. By positioning this topic from civil society, unprecedented online participation in the country was achieved. Furthermore, organisations were included in an advisory commission of the Ministry of Health on cancer [12,14].

Although civil society organisations on cancer in Chile have played a central role in positioning this topic on the public health agenda, there is limited information about them. It is unclear who they are, how they are organised, the objectives that guide their performance and how they materialize social participation in health. The only publicly available consolidated information on some organisations is contained in the National Register of Organisations Related to Health, a file created alongside the Law 20.850, which registers groups that have expressed their wish to be considered in the monitoring and recommendation commissions of this law. As a consequence, they do not necessarily reflect the diversity of groups currently involved in cancer advocacy.

The lack of knowledge about the diversity of groups working on cancer harms their possibilities to engage effectively with other actors to contribute to the public health challenges of this disease. Despite their historical relevance in Chile, they continue to be under-researched in public health and participation in health. This is consistent with the broader scenario of social organisations in health, which are often ignored as an essential part of social movements in the literature [3].

In this context, this study aimed to explore how civil society organisations in cancer are organised and represented, and how they collaborate with their relevant networks in the country. This was done through the perspectives of the organisations themselves, academics, politicians and decision-makers linked to the cancer theme in Chile. This research allows us to broaden perspectives on social participation in health and to address actors involved in the cancer issue that are not usually discussed by public health research, such as civil society organisations.

## Materials and methods

This methods section follows the consolidated criteria for reporting qualitative studies (COREQ) 32-item checklist, provided in the supplementary material.

### Study design

Qualitative multiple case study [15] that consists of obtaining a detailed understanding of the topic of study by gathering information from various cases. In this research, perceptions of cancer civil society organisations and their roles were addressed by multiple stakeholders’ perspectives: cancer civil society organisations, cancer policymakers, cancer decision-makers, and academics studying cancer. Each type of relevant actor was considered a case for this study, allowing for an in-depth understanding of the phenomena [15].

### Recruitment and participant selection

Theoretical and feasibility criteria were used to recruit and select participants [16]. The sample was purposive, as the researchers tried to achieve representativeness of the diversity of experiences of civil society organisations in cancer, as well as the diversity of other actors related to the topic.

The following inclusion criteria were used: i) Members of a civil society organisation working on cancer, ii) Decision-makers in the framework of the National Cancer Plan and Law, iii) Politicians with work related to the National Cancer Plan and Law, iv) Academics dedicated to research on cancer. All participants had to be older than 18 years old and have access to the internet to conduct interviews in digital format. The following exclusion criteria were used: i) Members of a civil society organisation that had been active for less than one year.

The sample intended to include registered and non-registered organisations in the National Register of Organisations Related to Health of the Ricarte Soto Law to reflect the diversity of organisations working on cancer-related topics in the country.

Recruitment was conducted online. Organisations listed in the registry mentioned above were contacted in a targeted way by searching online for their representatives’ contacts. Through this approach, 18 registered organisations were invited. Eight did not answer the invitation, and ten accepted the invitation to participate in the study. Researchers’ existing networks were used initially to recruit non-registered organisations, and then snowball sampling was activated among organisations. Information on groups from different areas of the country was also purposively searched on the internet and social media to ensure a range of other experiences in the sample. Through this method, ten non-registered organisations were invited. Four did not answer the invitation, and six accepted to participate in the study.

Politicians, academics, and decision-makers were purposively approached by e-mail based on their previous work in cancer-related fields. Four academics, five decision-makers, and three politicians were invited. All of them agreed to participate.

When recruiting, potential participants received information about the research’s aims, the topics considered in the interview, the institution to which the research team belonged, and the names and professions of the interviewers. It was pointed out that participating in the research was entirely voluntary and free of choice and that the organisations’ names would remain anonymous. None of the participants withdrew from the research during the interviews or afterwards.

Characteristics of participants of the study are shown in Table 1.

**Table 1:** Table of participants.

Identification code	Gender	Zone	Listed in the Register of Ricarte Soto Law
**Civil society organisations (n = 16)**			
E1, Registered organisation	Male	Metropolitan	Yes
E2, Registered organisation	Male	Metropolitan	Yes
E3, Registered organisation	Male	Metropolitan	Yes
E4, Registered organisation	Female	South	Yes
E5, Registered organisation	Female	Metropolitan	Yes
E6, Registered organisation	Female	Metropolitan	Yes
E7, Registered organisation	Female	South	Yes
E8, Registered organisation	Female	North	Yes
E9, Registered organisation	Female	Metropolitan	Yes
E10, Registered organisation	Female	South	Yes
E1, Non-registered organisation	Female	Metropolitan	No
E2, Non-registered organisation	Female	South	No
E3, Non-registered organisation	Female	North	No
E4, Non-registered organisation	Female	Metropolitan	No
E5, Non-registered organisation	Female	North	No
E6, Non-registered organisation	Female	North	No
**Decision-makers or health authorities (n = 5)**			
E1, Decision-maker	Male	South	–
E2, Decision-maker	Male	South	–
E3, Decision-maker	Female	North	–
E4, Decision-maker	Male	Metropolitan	–
E5, Decision-maker	Female	Metropolitan	
**Politicians (n = 3)**			
E1, Politician	Female	Metropolitan	–
E2, Politician	Female	Metropolitan	–
E3, Politician	Female	South	–
**Academics (n = 4)**			
E1, Academic	Male	Metropolitan	–
E2, Academic	Female	International	–
E3, Academic	Male	Metropolitan	–
E4, Academic	Female	Metropolitan	–

### Data collection

Semi-structured interviews were used as a data-gathering instrument. These interviews use a guide of topics related to the research aims. However, the specific questions and their formulation can vary depending on the respondents’ answers, context, and characteristics [17]. This technique allows the results to include the located and contextual aspects that can emerge in the conversation with the interviewed participant, enhancing the research depth and scope.

All semi-structured online interviews were conducted between 02 July and 08 September 2023. The interviews were conducted by AR, FV, CC, AO, and BC, who had previous experience in qualitative research and interviewing.

Although semi-structured interviews tend to use an open list of topics, a standard specific questions guide was built between the interviewers to establish a common approach to the interview process, still, according to the technique’s characteristics, researchers could adapt it to each interview depending on the context and topics the respondents chose to delve into.

The guide of topics was built around the study’s specific objectives and based on literature and expert comments, considering the themes of i) characterisation of organisations, ii) relationships and networks between civil society organisations in cancer, and iii) benefits and tensions that emerge when networking. The dimensions of interest and categories considered in the research are presented in Table 2. The standard questions guide can be found in the supplementary material.

**Table 2:** Dimensions and categories of interest.

Dimension of interest	Categories
Characterisation of organisations related to cancer in Chile	General description
	Functioning
	Role in cancer
Relationships and networking among cancer-related civil society organisations in Chile and with other cancer-related actors	Relations with organisations, institutions and people related to cancer in Chile
	Networks, alliances or collaborations between organisations, institutions and people related to cancer in Chile
Perception of the relationship with other organisations, institutions and people related to cancer in Chile	Benefits experienced in building relationships
	Difficulties experienced in building relationships

The interviews were conducted online using Zoom or another platform according to the participants’ preferences to facilitate the participation of organisations from different parts of the country. The audio file was recorded, lasting approximately 45 minutes. The information was stored in a private folder of the computer’s principal researcher with code access.

The information saturation was assessed after the research team had conducted a preliminary analysis of the data, resulting in the decision not to continue increasing the sample size of 28 interviews.

### Data analysis

The interviews were transcribed Verbatim to digital text files, anonymising sensitive information. Each interview and its respective transcript were reviewed by more than one researcher in the team. Content analysis [17] was conducted according to the categories in the interview script, admitting emerging categories and sub-categories from the participants’ narratives. The analysis was carried out by AR, FV, and CC, with subsequent review by AO, BC, and ME. Verbatim quotes included in this article were translated from Spanish by a native English speaker. The authors reviewed them to ensure the translations captured the original meaning.

**Fig 1:**
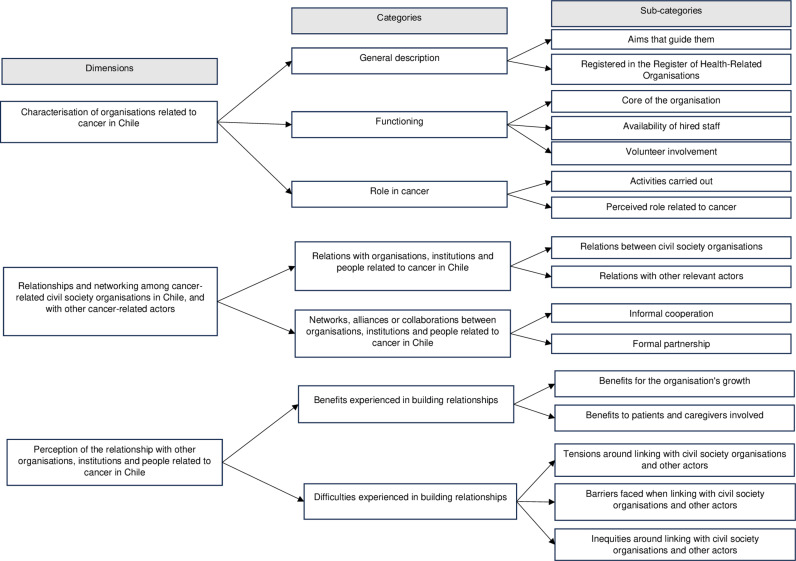
Coding tree.

The code tree built based on the content analysis is summarised in Fig 1.

### Qualitative scientific rigour

The rigour of the research was ensured by applying the quality criteria of qualitative research [17]. The specific ways to ensure methodological rigour were: (i) To ensure a logical correspondence between the problem to be investigated and the method used, in this case, to address the profiles and characteristics of civil society organisations through a methodology that would allow the vision of the actors involved to be captured, adopting a qualitative case study approach. (ii) The design of the sample was adequate, considering the different actors involved in the cancer issue in Chile from a perspective of social participation in health. (iii) Systematic description and detailed development of the research process, considering a detailed description from the collection to the analysis and writing of results. (iv) Triangulation of researchers, which was applied with the analysis and discussion of the team’s researchers from different disciplines of health and social sciences [17].

### Ethics

The study followed all ethical recommendations established by international consensus [18] and was approved by the Comité Ético Científico —Clínica Alemana—Facultad de Medicina—Universidad del Desarrollo (code 2023–60). A digital informed consent form was provided before the interview, explaining the research’s aims, details of participation, anonymisation of all sensitive information and names of the organisations that participated, and confidentiality in the data management. It stated that participants could withdraw at any time they wished. Participants gave their written consent to participate.

## Results

### Profiles of Civil Society Cancer Organisations (CSOs) in Chile

Based on what was described by the participants, the groups were characterised in terms of their motivations, objectives, activities and operating structures. The main driver for joining together with other people is their own experiences of cancer or those of close relatives, which lead them to identify opportunities for improvement in the pathway of this disease, which they address through different objectives. Some CSOs aim to assist patients’ needs through psychosocial and emotional support and navigation of the health care system. Others add to this aim the intention to provide education about cancer, address prevention in the general population and provide specific care for patients and their families. Some other organisations, commonly newly established, focus on particular areas of patient well-being, such as sports activities. Another type of civil society organisation aims to influence public policy and legislation on cancer. Among the last ones, there is a tendency to gather as many cancers as possible, while those focused on accompaniment, care, and assistance to needs tend to be cancer-specific organisations. This type of organisation tends to have a longer trajectory in social participation in health, with activities for five years straight or more.

*‘To be helpful, there is no profit motive here or anything else, it is just to help and to avoid a little bit that, when you are diagnosed, you feel alone. You don’t know where you are going and the feeling of anguish that loneliness generates is very dreadful, so it’s to ease the path a little or make it more, not happy and cheerful, because that would be unreasonable, but to make it a little more manageable’ (E6, Registered organisation).*

Decision-makers emphasise that the possibility of associating with other people is mediated by the existence of spaces for meeting with other patients and family members, which allows them to identify needs that affect them as a group. For example, the encounters in hospital treatment areas, such as the chemotherapy couch, stand out, allowing them to establish bonds and identify common problems.

In this context, they highlight the difficulty that this can have in small cities that do not have highly complex hospitals. In these cases, people move to other areas to receive treatment and medical consultations so they do not encounter other patients or family members from their areas at the time of treatment, which reduces meeting spaces and organisational capacities.

*‘[…] The oncological pathologies here do not have a surgical or treatment resolution; here, most of the oncological pathologies we prepare them so that they go to the Regional Hospital [in the regional capital] with everything ready, tidy, and as far as possible compensated. So there is no point where patients can meet and talk about what they are going through with this pathology, regardless of the type of cancer’ (E1, Decision-maker).*

Territorial differences were identified in terms of the aims of the organisations related to the concentrated institutional participation and decision-making spaces in the city of Santiago, the country’s capital. The participants pointed out that the groups formed in Santiago showed a greater interest in having a national scope and participating in decision-making and defining public policies. At the same time, those created in the regions focus on meeting local needs and contributing to the patients that live in those jurisdictions. Despite this distinction identified by participants, many regional groups seek to articulate with other national organisations in Santiago to broaden the possibilities for work on the themes they address.

*‘Well, without a doubt, everything costs twice as much in other regions, at least in my region. The organisations in Santiago are in Chile; we are absolutely isolated from the rest because, first of all, it is impossible to get here to the congress; if they manage to get here, one or two people come, if they do. It’s not such a participative subject for the same reason, because it’s very difficult for us to get here. Now everything is in Santiago, you have the ministries, you have the executive, the congress is very close [...]’ (E3, Politician).*

Based on their goals, CSOs implement a variety of activities. For assistance with needs, for example, fundraising activities are carried out to deliver food to the families. When they focus on community information objectives, activities include cancer awareness days, information workshops and discussion meetings. The groups that focus on political advocacy carry out awareness-raising, provide information and even legal assistance in some cases, as well as legislative discussion sessions and participation in round tables with the authorities.

The operating structure follows a similar trend among the organisations, even if they have different objectives. Depending on the CSO, it is generally structured around a core group of 3–5 people that acts as a formal or informal board. This group defines the objectives and guides the functioning of the organisation’s activities. Some more formal organisations have a small team of people hired to perform functions needed by the organisation, such as communications and research. Some organisations also have volunteers who participate in the activities, support groups and educational events.

From the perspective of academics and politicians interviewed, it is perceived that different leadership types are found among civil society organisations in cancer. It is mentioned that some groups put into practice leadership centred on individuals, while others focus on positioning the organisation as a collective entity beyond specific figures. The different forms of leadership can determine the organisation´s growth and impact, as those with personal solid leadership connected to relevant figures in the public debate acquire greater visibility and achieve more effective actions to address their needs.

*‘Whereas in other cases you don’t see the corporation so much, you don’t see the group so much, but you see the leader and you feel that, if that leader were not there, the organisation would not be sustained, that is the differentiation I am making, maybe not with a good label, but that is what I mean, let’s say, that is the difference. Some depend on the leader and others depend on the institution’ (E1, Academic).*

In addition to the characteristics described above, the time the organisations have been active was also considered to describe the main profiles of civil society groups working on cancer in Chile. This was included because there were similarities between the functioning time of the groups and their aims. Long-established organisations were considered when they had been active for five years or more. Growing organisations were considered when they had been active for more than two years but less than five, and organisations were considered newly established when they had been functioning for more than one year.

Long-established organisations usually focus on raising awareness and influencing public policy and decision-making in cancer. They tend to have a national scope, gather various diagnoses, and establish working linkages with health professionals, politicians, and decision-makers to influence public policy. They are usually registered in the Registry of Organisations to participate in institutional discussion spaces.

*‘We have many patients and foundations behind us and the idea is to do something, each foundation itself has its own, there are some that are more assistance-oriented, paternalistic, other groups that are purely patients, but in [name of group] the idea is to try to raise the topic of the problem that in this case we are talking about the national cancer plan, the DAC, the commission, so we have a map drawn up and the idea of being able to register was to be a relevant actor and for us to have relevance’ (E2, Registered organisation).*

Growing organisations commonly aim to inform, raise awareness, and support families and patients. They tend to focus on specific areas, particularly when they arise in a region outside the metropolitan area. They address multiple needs in terms of physical and mental health and the socio-economic situation of patients. Some are registered because they perceive this allows them to be formally recognised as a group.

*‘Basically, to make the cancer experience a little less hard, attending mainly to aspects of accompaniment and those areas that the GES does not cover’ (E1, Non-registered organisation).*

Newly established organisations usually focus on specific areas of patient well-being, such as sports activities. They are often active at the local level, though their network is at national level. In addition to focusing on well-being activities, they raise awareness and support patients and families. They are not usually listed in the Register, as some see it as a subsequent step, when the organisation becomes more mature. In contrast, others report a lack of knowledge of the usefulness of being listed in the Register.

*‘And our vision is to be a national reference in the promotion of physical activity as part of the recovery and wellbeing of women breast cancer survivors [...] Through our activities and values, we hope to be a source of hope and strength for women on their journey to recovery and a full life’ (E5, Non-registered organisation).*

Participants recognise that these three main profiles may be fluid over time, depending on the definitions and actions taken by their members.

From the perspective of politicians, decision-makers and academics interviewed, the diversity of roles and objectives among civil society groups is appreciated. They argue that this enhances the debate and the possibilities of improving public health in cancer by parallel targeting different areas of need for patients and carers. Therefore, it is important to value this diversity of objectives and not impose it as a goal for all organisations to advance towards decision-making.

‘*Perfect, look there is something essential that we try to promote and that is that not all patient organisations have the mission of representativeness, there are small patient organisations that are born with a very specific vision, very limited and that is how they are going to end up and we are nobody to say that they have to be different’ (E2, Academic).*

### Networking among civil society organisations in cancer in Chile

Participants identified that the closest relationships organisations have is with other CSOs in cancer. In second place, they mention health authorities and health institutions, and in third place, health professionals. The private sector, academia and the pharmaceutical industry appear less frequently.

The connections with these actors are established through two main mechanisms: i) informal connections and ii) formal collaboration agreements. The narratives clearly show that both mechanisms of connection are implemented with the different actors, even though informal links are predominantly mentioned, particularly in newly established organisations. Although formal links are prioritised for funding, funding is also obtained informally from the private sector. The links generated are presented in Table 3.

**Table 3:** Networking among civil society organisations in cancer in Chile.

Types of linkages	Actors with which organisations interact	Aims of the connection	Quotes
Formal: sponsorships, e-mail letters or collaboration agreements signed at institutional level.	Other civil society organisations	Transmission of information. Collaborative working agendas. Transparency of activities.	*‘Yes, we link up mainly because we have actions in common that have to do with patients, both adults and children, and also with actions that we do for prevention, education, so that we can share our actions and activities, we have repeated them several times during the year’ (E1, Registered organisation).*
	National Health Authority and Health Institutions	Influence on decision making. Obtaining information. Seeking solutions to problems.	*‘With the ministry we have also participated, but I am not so clear on what, because I have participated in clinical guidelines on breast cancer or mental health’ (E5, Registered organisation).*
	Private sector	Funding for the implementation of activities. Agreements for the provision of services.	*‘[private clinic name] [...] Access to health care for samples and exams at cost price for Fonasa A (Public Insurance Fund) patients, for example, who suffer the most’ (E3, Registered organisation).*
	Pharmaceutical industry	Funding for activities.	*‘It is a close relationship unlike other patient groups we don’t hide them, on the contrary, we support them, because in one way or another they are what sustain us with the events or stuff that we have to do’ (E2, Registered organisation).*
	Academy	Sponsorship of activities. Participation in academic seminars on cancer. Participation in meetings to identify critical issues in cancer.	*‘We have had some involvement with some academies, we have even been involved in some postgraduate training, giving scholarships to certain universities and their students who are physicians who are going to specialise in oncology, and the foundation has given scholarships by paying for these training courses’ (E1, Registered organisation).*
Informal: personal contacts, based on previous acquaintance or individual wills.	Other civil society organisations	Guidance and counselling. Sharing experiences. Common activities.	*‘We already have two “babies”, we have the foundation [civil society organisation], which are from Araucanía region, and we have [civil society organisation] which is also a group of breast cancer patients from Chillán that we were able to accompany and help in the process before they were formed in this case as a functional and territorial organisation, and I think that’s the beauty of it’ (E3, Registered organisation).*
	Local Health Authority and Health Institutions	Obtaining information. Seeking solutions to problems.	*‘The [Regional Hospital]. Because they, from the beginning, have supported us [...] Yes, yes, so far it is unconditional’ (E2, Non-registered organisation).*
	Healthcare professionals	Access to health information. Access to institutional health care.	*‘[A specific healthcare professional] He has been very concerned about us and has guided us in the sense of health, and he also trains us in technology, because we don’t know how to see the QR code, for example, that we have to take something out, and he is very good at that. That’s why it’s important for us, besides, he has been a good guide’ (E2, Non-registered organisation).*
	Private sector	Funding for activities.	*‘The collaborators are people who come to our organisation, and they are the ones who give us support. These collaborators include companies, trade unions, schools, mothers‘ centres and other groups that provide us with support in the form of merchandise, cleaning supplies, school supplies, and what we give to families with oncological children is what we receive from these collaborators’ (E8, Registered organisation).*
	Academy	Sponsorship of activities. Participation in academic seminars on cancer. Participation in meetings to identify critical issues in cancer.	*‘I try to go to all the webinars to which they invite me because you learn, and then you replicate this with your patients, you replicate it with your team, you share the information and [academic name] has been, besides, it’s very lovely to work with her’ (E3, Registered organisation).*

### Benefits that arise from establishing links from civil society organisations in cancer

The representatives of the civil society organisations participating in the study reported two main benefits of linking up with other actors: contributions to the organisation’s growth and direct benefits for patients and caregivers who participate in and collaborate with the organisation. The contributions to the organisation’s growth are obtained mainly through formal and informal relationships between groups, where they share helpful information that allows them to improve their work, particularly in supporting patients and caregivers. For example, they report sharing information about recommended health professionals, particularly outside the capital, and about shelters in other regions to access treatment. These relationships allow them to overcome the lack of information in the regions about access to health care and, in some cases, to avoid traveling to Santiago. Yet, it is worth mentioning that oncology specialists and more complex treatments are concentrated and delivered in the capital, which means that commuting is required in many cases depending on the patient’s needs.

*‘It has happened to us, for example, people from the south who are in the organisation and know someone, know a doctor in their region and say ‘hey, I have my doctor here and I told him I was in the organisation’ and the doctor has contacted us, and now we know, when someone consults us about a doctor, we will send him to that doctor, because we know he is close to his commune or his region. That has been like the big benefit’ (E4, Non-registered organisation).*

Furthermore, the respondents mentioned the creation of mutual support networks to access information on applications for funding from public institutions, because the access to this information is cumbersome. They also highlight the dynamics of accompaniment between organisations, where long-established groups with a focus on decision-making act as guides or advisors to growing groups with a local scope based on their own previous experiences.

On the other hand, connecting with the private sector generally provides direct benefits for patients and carers. They mention typically obtaining arrangements for low-cost testing and funding for the organisation’s activities. Supporting testimonies can be found in Table 2.

### Tensions, barriers and inequities that emerge when building links from civil society organisations in cancer

Despite the benefits identified, there are perceived tensions, barriers, and inequities in the interactions between organisations and with other actors. Table 4 summarises these tensions, barriers, and inequities.

**Table 4:** Tensions, barriers and inequities that emerge when building links from civil society organisations in cancer.

Results of linking	Specific category	Quotes
Tensions	Competitive dynamics	*‘[...] There were disputes between organisations and arguments, like “are you from this group or are you from that group”, so [the name of the organisation] was new, it didn’t have those problems. We arrived and, all of a sudden, we realised that there were a lot of organisations and they were all fighting, like there were sides, so it was very difficult to work when there are sides, it’s like ‘if you’re friends with him, you’re not friends with us’, this dispute was a bit sterile for us, to be honest, and very childish’ (E2, Registered organisation).*
Barriers	Limited professionalisation of communication channels	*‘I think that this has been the main difficulty, I don’t know, you are in [federation of groups], you are new and hey, I need to send an email to everyone, “Oh, we don’t know where the list is”, look for it and it arrives, but I tell you that we need to be a bit more serious, to have more professional tools’ (E1, Non-registered organisation).*
	Lack of knowledge about contact channels with other actors	*‘I think it would be very important to have, but I don’t know about it, entities that can build bridges between companies that are looking for serious institutions, especially now, that are looking for serious institutions linked to certain causes and those organisations that represent those causes, but I would say that they are more obstacles or barriers’ (E5, Registered organisation).*
	Shift in focus and working styles as government changes	*‘But it is complex to work with institutions, because you depend on the public policies that are trending, or on the person who is in charge, who starts everything from scratch, every time they change the person in the position. And the bureaucracies that also go in between, a decision, someone may really want to change the world, but they have to go through 12 people, plus 400 legal analyses, before they can make a decision’ (E9, Registered organisation).*
Inequities	Differentiated visibility depending on cancer type	*‘I see it as complex, and unequal. I think some cancers, right, like breast cancer, or this GIST (gastrointestinal stromal tumour), right? [...], which has achieved visualisation, events, widespread support from society, many drugs and a lot of money in different coverage, in GES, additional coverage by Ricarte Soto in the case of breast cancer and, furthermore, they are included in DAC recommendations with last line therapies, and from that point of view it is good, and I am pleased that there is participation, however, I think there is an inequity, because the other cancers, which do not have leaders, right? because they affect poorer people, who don’t even have the capacity to group together, they are not visible, right?’ (E4, Academic).*
	Territorial inequities	*‘It isn’t easy, it’s like we are here, below, and they are above, in the Olympus, and it is very complicated to reach them, and if we want to reach them, there is always an intermediary and the intermediary is never going to say things the way we would like it to reach the right person’ (E2, Non-registered organisation).*

Tensions relate to the dynamics of competition between organisations, which, according to the interviews, respond to the clash of egos between leaders and groups, where different actors seek to position themselves as the only organisation with the most significant influence on cancer matters. In some cases, this leads to negative judgements and criticism between organisations about funding obtained by other organisations and their collaborations, limiting the possibilities for widespread cooperation between different groups. From the perspective of some civil society interviewees, this tension scenario could be explained by profound differences in working styles and focus between groups. At the same time, the existence of limited resources to fund civil society initiatives would promote rivalries and competition, as groups would have to fight with each other for survival and funding constantly.

*‘So, as resources are scarce, I have been surprised that sometimes it is difficult to collaborate because resources are scarce, so there is a certain competition’ (E1, Non-registered organisation).*

*‘Also with peers, with peers, I mean with other organisations that I know too, this is very curious because egos are generated in these things, it’s like the old “conventicle”, so people like you say “look, this other guy made a logo, who paid for the logo, where did he get the money, who designed it”’ (E1, Registered Organisation).*

While the research participants acknowledged that these dynamics are prevalent among the groups and a recurring theme in the national landscape, none of them claimed sole responsibility for these behaviours or dynamics.

In terms of barriers, the dynamics of competition are positioned as a barrier mainly for new groups, who say they are unaware of the friction between organisations and are facing difficulties in knowing how and with whom to relate to grow. On the other hand, the fact that there is limited professionalisation of the forms of communication and the procedures carried out by the organisations is mentioned as a barrier to establishing relations between groups. This reality hinders and slows the possibility of establishing joint collaborative relationships and learning about the work, objectives, and activities of similar groups.

A critical barrier, as perceived by the participants, is the lack of knowledge within civil society organisations about how to initiate collaboration with institutions such as private companies, the Ministry of Health, and other public bodies. As a result, the connections they form with other actors largely depend on personal contacts and, to a lesser extent, on chance encounters where they stumble upon information online.

In terms of the barriers perceived by the organisations to engage with public institutions, the main obstacle mentioned was the constant changes in their goals and ways of operating with each new government. Participants even recognise changes in strategic outlooks, each implemented by the specific officials in charge. At the same time, the growing bureaucracy that affects decision-making by the country’s health authorities was identified as a barrier. This shows that communication dynamics are person-dependent on the part of both CSOs and the State and are marked by a lack of mutual information.

*‘But it is complex to work with institutions because you depend on the public policies that are in fashion, on the person in charge, who starts everything from scratch, every time they change the people in charge. The bureaucracies also go in between a decision; someone may really want to change the world, but they have to go through 12 people, plus 400 legal analyses, before they can make a decision’ (E9, Non-registered organisation).*

In terms of inequities, the possibility of establishing links with other actors is also tied to the visibility that each type of cancer acquires, allowing them to connect better with different groups and health institutions. This would respond, on the one hand, to leadership styles. As noted above, those who have strong leadership and are well-connected with authorities and political figures find easier to give visibility to their cancer and to the political interest that a disease may arouse at a given time.

*‘I believe that the political arena, when it learns of a great need, with numbers and arguments, is what catapults things to happen [...] the patient groups that see an opportunity to move together, right? and you have the support at least on paper of the public world, at least of the ministry, probably that generates spaces for training, for growth with resources generated by these other parties, but that’s it’ (E3, Academic).*

Furthermore, the territorial differences in a country like Chile, which has a concentration of decision-making in the capital and the health institutions that provide more complex care, determine inequities in networking opportunities. This affects to groups residing in more remote regions, which is exacerbated in rural areas.

Finally, the politicians, decision-makers and academics who participated in this study, emphasized that organisations represent a particular profile of patients and carers interested in linking up with others and generating support networks. This would leave out people who do not actively seek to connect with others, which may generate inequities in the benefits attributable to social participation. Therefore, it is relevant to take a broad view of cancer’s needs and consider what is happening in spaces where social participation has not been materialized.

*‘At the end of the day, this support ends up favouring those who have this, let’s say, positive predisposition to actively seek support networks, etc., and not the other profile of a person, who, probably, due to lack of knowledge, or personality profile or whatever, do not manage to reach these groups, right? [...] So, from that point of view, I think it produces inequity, it’s strange because deep down it’s like, social organisation is supposed to be more transversal, but I think it’s not as transversal as it should be’ (E4, Academic).*

## Discussion

This qualitative study explored the characteristics of civil society cancer groups in Chile based on the narratives of the organisations, academics, politicians and decision-makers working on cancer in the country. This study allowed us to identify profiles of the organisations based on their objectives, activities and ways of functioning and explored the relationships they establish with other actors such as public health institutions, private sector, academia and pharmaceutical industry. We also identified benefits obtained through these relationships and tensions and barriers of different types, depending on the actor and the kind of relationship established. This is crucial for strengthening social participation in health through knowledge of the involved actors and the tensions underlying their interactions.

The results reveal that civil society organisations in cancer are a heterogeneous group. They have various motivations, for example, to meet the needs of cancer patients and their families, to provide support and guidance to those suffering from this condition, to generate activities for physical and psychosocial well-being, and to advocate for legislative and regulatory advances. The roles that cancer organisations are reportedly playing are consistent with those historically performed by health organisations in Chile and other critical areas of need for the population [4,6,14,19]. At the regional and global level, there are diverse experiences where civil society plays relevant roles in highlighting needs and contributing with insights to the management and accompaniment of various chronic and transmissible diseases [20–23]. In the specific case of cancer, the literature shows that organisations at the regional and global levels have played similar roles to the groups in Chile, making visible opportunities for improvement in care and social support for patients and carers living with this disease and generating recommendations for decision-makers [5,14,24,25].

We also observed that the actions carried out by cancer organisations pursue different objectives, which may embody different ways of practising social participation in health. Those who focus on meeting needs and generating well-being for patients and caregivers would be exercising a form of involvement close to what is identified in the literature as self-management in health [3]. In contrast, those who seek to influence decision-making would be putting into practice social participation in health linked to institutionality. This approach predominates in health systems [2]. These different approaches to social involvement may go unnoticed by the health authorities, given the mere consultative role historically given to social health organisations in Chile [10,26].

The results reveal that the organisations do not act independently to achieve their objectives. Instead, they tend to form alliances with other groups and social actors to advocate for their interests. Despite this variety of approaches among cancer organisations, their aims seem to allow them to address different fronts of need for cancer patients and their families in parallel.

Our findings also indicated that a series of tensions, barriers and inequities are evident in the network of relationships between cancer CSOs, mainly linked to competitiveness for funding, visibility, and differences about connections with health authority figures and public figures, including the political world. The study also unveiled territorial inequities by type of cancer. Representatives highlight that, despite the different efforts for cooperation among patient´s groups, an underlying competitiveness and tension persist in understanding and exercising social participation in health, especially in a scenario with limited participation mechanisms.

Based on these tensions, social participation in health ends up being exercised unequally among cancer groups in Chile, both in terms of decision-making and liaison with authorities, as well as in the deployment of these groups’ capacities to accompany patients and remedy their multiple needs. Indeed, the potential to transform the living conditions of patients and their families is limited because actions performed by civil society are not visible. Engaging communities across the continuum of care is one of the critical elements established by the Alma-Ata declaration to promote people’s health [27] effectively. Yet, according to the literature, it is one of the least worked principles of this declaration in the long term [28]. This leads to missed opportunities to advance the public health needs of the population collaboratively between institutions and civil society, a strategy that could impact reducing health inequities [23].

The strengths of this study include its qualitative approach, which allowed us to produce an initial characterisation based on the visions and narratives of the patient´s groups and other relevant actors who work in the field of cancer in Chile. This exercise is particularly relevant because the country does not have a specific register of the cancer organisations of civil society, so this initial characterisation can contribute with an in-depth description of the dynamics, activities and roles that cancer-centred civil society is developing, which may be overlooked by institutions and authorities. Additionally, in the context of the implementation of the National Cancer Plan and National Cancer Law, both with social participation in health as a central pillar [12,13], this characterisation of civil society can contribute to establishing protocols of involvement appropriate to the organisations’ characteristics and needs, making its results directly applicable to social participation in cancer in Chile.

Additionally, this study can be helpful to social participation in health at the global level and beyond the specific dynamics of the cancer topic. The World Health Organisation (WHO) recognises the relevance of collaborating with CSOs in health [29]. To achieve this goal, in the early 2000s, the need to collect systematic evidence on the role of CSOs in health and the relevance that the public health sector can understand CSOs and their dynamics was already established [30]. Recent literature suggests that participation requires legal frameworks and registering effective systems that can attract CSOs but also reflect them [31,32]. Recognition capacities, consisting of understanding the context, actors, and key stakeholders present where the organisations are immersed, are suggested as prerequisites to enhancing and strengthening social participation [32]. Also, a recent study on participatory governance in health concluded that the design of relevant reforms to the health system requires a localized understanding of civil society in their specific contexts [33].

This research helps address these global goals and needs of social participation in health, contributing with its methods and results to enrich the literature in the field. It offers a novel perspective on understanding CSOs, including the tensions, barriers, and inequities that emerge when civil society organisations relate with each other and other actors. This allows a deep understanding of the local context, which, as seen before, should be a mandatory first step to developing social participation in health and other matters, independent of the circumstances and conditions. The dimensions and categories of interest considered in this qualitative study used to characterise CSOs in cancer can be adapted to different contexts by public institutions, academics and even civil society itself to identify the characteristics of civil society in that specific context in depth.

However, the study has limitations, including a small number of mainly urban and capital-based organisations, and it did not disclose the organisation’s names, which may restrict the study’s potential for broader use. Future studies could explore in more detail the profiles of organisations associated with different types of cancer and carry out territorial comparisons between rural and urban areas and between countries to enrich knowledge about social participation in health.

These results emphasise the relevance of recognising cancer CSOs as essential actors in public health in Chile who contribute to improving the quality of life of patients and their families. Given their diversity of competencies, it is crucial to involve them in formulating and implementing comprehensive responses to cancer challenges to maximise opportunities for progress in this field. Building knowledge about these actors from academia and institutions and setting common goals will strengthen social participation in cancer from an inclusive perspective at the local and regional levels. Also, shedding light on the organisations that emerge around the cancer illness contributes to broadening the perspectives around this disease, showing how people re-signify their illness as a collective experience. In addition, producing specific information on the characteristics of these organisations offers an innovative approach to the study of social participation in health. Approaching it from the description and analysis of the actors involved allows us to address a gap in the literature on social movements in health.

## Supporting information

S1 File CORE-Q checklist. (DOCX)
