# Supplementary material for: Exploring the characteristics of cancer-centred civil society organisations in Chile: A qualitative study
Source: PLoS One. 2025 May 16;20(5):e0315589. doi: 10.1371/journal.pone.0315589 (PMC12084054; doi:10.1371/journal.pone.0315589)
Supplement: S1 File — (DOCX) [file pone.0315589.s001.docx]

# **Exploring the Characteristics of Cancer-Centred Civil Society Organisations in Chile: A Qualitative Study**

**Supplementary File 1:** CORE-Q checklist of the research “Exploring the Characteristics of Cancer-Centred Civil Society Organisations in Chile: A Qualitative Study”

| Item | Topic | Guide Question | Descriptions | Report on (page/table) |
| --- | --- | --- | --- | --- |
| **Domain 1: Research team and reflexivity** | | | | |
| *Personal characteristics* | | | | |
| 1 | Interviewer/facilitator | Wich author/s conducted the interview? | AR, FV, CC, AO, and BC, all with previous experience in qualitative research, conducted the interviews. | Report on page 7 of the manuscript. |
| 2 | Credentials | What were the researcher’s credentials? | All the researchers had previous experience in qualitative research and came from social sciences or health disciplines related to the topic of study. | Supplementary material N°2. |
| 3 | Occupation | What was their occupation at the time of the study? | The occupations of the researchers on the study team are described in the Supplementary Material. | Supplementary material N°2. |
| 4 | Gender | Was the researcher male or female? | Five females and 1 male. The genders and initials of all the researchers are described in Supplementary Material. | Supplementary material N°2. |
| 5 | Experience and training | What experience or training did the researcher have? | All the participants had previous experience in research. The specific experience and training of each researcher is described in Supplementary Material | Supplementary material N°2. |
| *Relationship with participants* | | | | |
| 6 | Relationship established | Was a relationship established prior to study commencement? | The researchers informed all potential participants of the study objectives and the topics considered in the interview, and it was pointed out that participating in the research was entirely voluntary and free of choice. | Pages 5 and 6. Section: Recruitment and participant selection. |
| 7 | Participant knowledge of the interviewer | What did the participants know about the researcher? (e.g., personal goals, reasons for doing the research) | Potential participants were informed of the main researcher's name, place of work, and reason for conducting the research. | Pages 5 and 6. Section: Recruitment and participant selection. |
| 8 | Interviewer characteristics | What characteristics were reported about the interviewer/facilitator? E.g., bias, assumptions, reasons and interest in the research topic? | Potential participants knew the interviewer's name, place of work and reason for doing the research. Also, they were informed of the reason and importance of this research. | Pages 5 and 6. Section: Recruitment and participant selection. |
| **Domain 2: Study design** | | | | |
| *Theoretical framework* | | | | |
| 9 | Methodological orientation and theory | What methodological orientation was stated to underpin the study? E.g., grounded theory, discourse analysis, ethnography, phenomenology, content analysis. | We employed a content analysis in this study. | Page 5. Section: Type of study. |
| *Participant selection* | | | | |
| 10 | Sampling | How were participants selected? E.g., purposive, convenience, consecutive, snowball. | We used a purposive sample to select study participants based on the theoretical criteria established and their availability and willingness to participate. | Pages 5. Section: Recruitment and participant selection. |
| 11 | Method of approach | How were participants approached? E.g., face-to-face, telephone, mail, email. | Recruitment was conducted online. Depending on the availability of the data on the Internet, participants were contacted through email, telephone, or social media.  The research team recruited participants through social networks, civil society organizations, and snowball techniques. | Pages 5. Section: Recruitment and participant selection. |
| 12 | Sample size | How many participants were in the study? | There was a total of 28 participants in the study. | Page 5. Section: Recruitment and participant selection. |
| 13 | Non-participation | How many people refused to participate or dropped out? Reasons? | Participation in the in-depth interviews was voluntary, and no participant declined to participate. | Page 5. Section: Recruitment and participant selection. |
| *Setting* | | | | |
| 14 | Setting of data collection | Where was the data collected? E.g., home, clinic, workplace. | The data was collected online to facilitate the participation of people from different areas of the country. | Page 7. Section: Data collection. |
| 15 | Presence of non-participant | Was anyone else present besides the participants and researchers? | No one else was present besides the researcher and participants. |  |
| 16 | Description of sample | What are the important characteristics of the sample? E.g., demographic data, date. | Participants’ sociodemographic data are described on Page 6. Section: Recruitment and Participant Selection. Presented in Table 1. | Page 5. Section: Recruitment and Participant Selection. Presented in Table 1. |
| *Data collection* | | | | |
| 17 | Interview guide | Were questions, prompts, guides provided by the authors? Was it pilot tested? | The research team provided the dimensions of interests and categories that helped building the interview script. It is on page 7. Section: Data collection. It is presented in Table 2.  The interview script is attached on supplementary file N°3. | Page 7. Section: Data collection. Table 2.  The interview script is attached on supplementary file N°3. |
| 18 | Repeat interviews | Were repeat interviews carried out? If yes, how many? | There were no repeat interviews carried out in this study. |  |
| 19 | Audio/visual recording | Did the research use audio or visual recording to collect the data? | The audios of the interviews were recorded via Zoom, which were later transcribed verbatim, and checked backwards for accuracy for the research team. | Page 7. Section: Data collection. |
| 20 | Field notes | Were field notes made during and/or after the interview or focus group? | N/A |  |
| 21 | Duration | What was the duration of the interview or focus group? | The interviews took approximately 45 minutes. | Page 8. Section: Data collection. |
| 22 | Data saturation | Was data saturation discussed? | The information saturation was assessed after interim data analysis by the research team. | Page 8. Section: Data collection |
| 23 | Transcripts returned | Were transcripts returned to participants for comment and/or correction? | More than one research team member confirmed each audio and transcript of the interviews. | Page 9. Section: Data Analysis |
| **Domain 3: analysis and findings** | | | | |
| *Data analysis* | | | | |
| 24 | Number of data coders | How many data coders coded the data? | Three researchers on the team coded the data. |  |
| 25 | Description of the coding tree | Did authors provide a description of the coding tree or codebook? | The manuscript contains the information about the coding tree for this study, in Fig. 1. | Page 8. Section: Data Analysis. Fig. 1. |
| 26 | Derivation of themes | Were themes identified in advance or derived from the data? | Themes identified in advance according to the categories considered on the research, and also emergent themes from the interviews where identified. | Page 8. Section: Data Analysis. |
| 27 | Software | What software, if applicable, was used to manage data | No software was used. |  |
| 28 | Participant checking | Did participants provide feedback on the findings? | Participants received an executive resume of the findings via e-mail. Participants did not give feedback. |  |
| *Reporting* | | | | |
| 29 | Quotations presented | Were participant quotations presented to illustrate the themes/findings? Was each quotation identified? e.g., participant number. | Representative quotes and their respective codes are presented in the Results section of the manuscript. | Pages 9-21. Section: Results. |
| 30 | Data and findings consistent | Was there consistency between the data presented and the findings? | We demonstrate consistency between the data presented in the results section and the interpretation of findings in the discussion section. |  |
| 31 | Clarity of major themes | Were major themes clearly presented in the findings? | We presented all major themes according to the objectives and methods of the study. |  |
| 32 | Clarity of minor themes | Is there a description of diverse cases or discussion of minor themes? | In the Results section, we provide a diversity of quotes according to the different perspectives of participants. | Pages 9-21. Section: Results. |

**Supplementary File 2:** Research Team Characteristics and Qualifications

| **Author** | **Characteristics and Qualifications** |
| --- | --- |
| AR | Female, Sociologist (Universidad de Chile). Diploma in Design, Management and Evaluation of Projects of Public Interest (Universidad de Chile). Currently pursuing a Master's in Public Health (Universidad de Chile). Researcher in Centre for Global Intercultural Health (CeSGI), Facultad de Medicina - Clínica Alemana Universidad del Desarrollo y Facultad de Psicología Universidad del Desarrollo. Research interests include public health, inequities, patient journeys, patient participation. |
| FV | Female, Social Anthropologist, MSc (c) in Public Health (Universidad de Chile). Diploma in Quantitative Methodologies (Universidad Diego Portales), and diploma in Formulation and Evaluation of Social Projects focused on the territory and the community (Pontificia Universidad Católica de Chile). Researcher in Centre for Global Intercultural Health (CeSGI), Facultad de Medicina - Clínica Alemana Universidad del Desarrollo y Facultad de Psicología Universidad del Desarrollo.Research interests include anthropology and health, public health, inequities, patient participation, cancer and rare diseases. |
| CC | Female with a Master of Management in Primary Health Care, and PhD© for the Doctor in Sciences and Innovation in Medicine program. Qualitative, multi-methods, and epidemiology research training. Her research interests include cancer, rare diseases, inequities, global health, and patient participation. |
| BC | Female Nurse-midwife (2002, PUC Chile), diploma in university teaching (2004, PUC Chile), Master in Epidemiology (2008, PUC Chile) and PhD in Health Sciences (mention in social epidemiology) from the University of York, England (2011). Professor of social epidemiology and director of the Centre of Global Intercultural Health, Facultad de Medicina - Clínica Alemana Universidad del Desarrollo y Facultad de Psicología Universidad del Desarrollo. Visiting scholar at the Department of Health Sciences at the University of York. Member of Lancet Migration for Latin America, board member of the Chilean network of research on health and migration RECHISAM. Former Vice-President of the Chilean Society of Epidemiology between the years 2014-2017. National and international consultant of health equity, health of migrants and implementation science in socially and culturally diverse communities. Advisor to WHO, PAHO, the Ministry of Health and the Ministry of Social Development of Chile, and to various public and private institutions. Research lines: social inequities in health (2005 to date), health of international migrants (2008 to date) and participation of patients in decision-making on health coverage (2016 to date). Editor of 8 academic books and over 20 publicly available research reports and policy briefs. Participation in more than 60 research projects in Chile and abroad and has more than 170 scientific publications. |
| AO | Female Social Anthropologist. She has a master's degree in Gender and Cultural Studies from the University of Chile, and a doctorate in Social and Cultural Anthropology from the University of Barcelona. Associate Professor and Executive Director of the Centre for Global Intercultural Health (CeSGI), Facultad de Medicina - Clínica Alemana Universidad del Desarrollo y Facultad de Psicología Universidad del Desarrollo. She is an expert in health, gender and intercultural issues. Research focuses on health anthropology, adolescent and youth sexual and reproductive health, patient participation in health decision-making, and qualitative methodologies. Principal investigator of various research projects, including: Fonis Project #SA15I20040; Fonis Project #SA19I0091; Fondecyt #11190701. |
| ME | Male Medical Doctor and Master in Epidemiology both from Pontificia Universidad Católica de Chile; a Master in Biostatistics from Universidad de Chile, and Master of Science and a PhD in Health Economics, both from University of York in the UK. Associate Professor in the School of Public Health at Pontificia Universidad Católica de Chile. Editor-in-chief of Value in Health Regional Issues and Chair of the Latin America HTAi Policy Forum. Consultant for the Interamerican Bank of Development and the World Bank; and Chief Scientific Officer (CSO) Epsilon Research. His research is focused on health technology assessment, methodological and applied economic evaluation in healthcare, priority setting, and stakeholder participation in decision making about coverage. |

**Supplementary File 3:** Interview script.

| **Participants** | **Dimension of interest** | **Categories** | **Interview script** |
| --- | --- | --- | --- |
| Civil society organisations | Characterisation of organisations related to cancer in Chile | General description | Could you briefly describe your organisation, including its mission and vision?  Is your organisation registered in the national register of health-related organisations? Why or why not? |
|  |  | Functioning | How does your organisation function in terms of structure?  How does the participation of your members work? |
|  |  | Role in cancer | What is the role of your organisation in cancer-related topics in Chile?  What activities does your organisation carry out to address cancer-related challenges? |
|  | Relationships and networking among cancer-related civil society organisations in Chile and with other cancer-related actors | Relations with organisations, institutions and people related to cancer in Chile | Does your organisation have links with other cancer-related organisations in Chile? How would you characterise these links?  Does your organisation have links with other cancer-related actors in Chile? How would you characterise these links? |
|  |  | Networks, alliances or collaborations between organisations, institutions and people related to cancer in Chile | Are there networks, partnerships or collaborations between organisations? How would you characterise them?  Are there any networks, alliances or collaborations with other actors? With which actors? How would you characterise them? |
|  | Perception of the relationship with other organisations, institutions and people related to cancer in Chile | Benefits experienced in building relationships | What benefits have you experienced from establishing these links? |
|  |  | Difficulties experienced in building relationships | What difficulties have you experienced in establishing these links? |
| Decision-makers, Politicians and Academics who relate to cancer in Chile | Characterisation of organisations related to cancer in Chile | General description | What knowledge do you have about cancer-related civil society organisations in Chile? |
|  |  | Functioning | What is your perception of the profiles of the organisations in terms of their mission and the way they operate?  What activities or initiatives have you observed in these organisations concerning cancer in Chile? |
|  |  | Role in cancer | Do you think these organisations are essential in addressing cancer in Chile? Why? |
|  | Relationships and networking among cancer-related civil society organisations in Chile and with other cancer-related actors | Relations with organisations, institutions and people related to cancer in Chile | Have you observed any interaction or collaboration between cancer organisations in Chile? Could you describe them?  Have you observed any interaction or collaboration with other relevant actors from cancer-related organisations in Chile? If so, could you describe them? |
|  |  | Networks, alliances or collaborations between organisations, institutions and people related to cancer in Chile. | Have you observed the existence of networks or alliances that facilitate the joint participation of these organisations? Could you describe them?  Have you observed the existence of networks or alliances that facilitate linkages with other actors? Could you describe them? |
|  | Perception of the relationship with other organisations, institutions and people related to cancer in Chile | Benefits experienced in building relationships | What benefits have you observed in the relationships that organisations build? |
|  |  | Difficulties experienced in building relationships | What difficulties have you observed in the relationships that organisations establish? |
